# Flow physics and mixing quality in a confined impinging jet mixer

**DOI:** 10.1063/5.0002125

**Published:** 2020-04-02

**Authors:** Yue Hao, Jung-Hee Seo, Yizong Hu, Hai-Quan Mao, Rajat Mittal

**Affiliations:** 1 Mechanical Engineering, Johns Hopkins University, Baltimore, Maryland 21218, USA; 2 Biomedical Engineering, Johns Hopkins University, Baltimore, Maryland 21287, USA; 3 Institute for NanoBioTechnology, Johns Hopkins University, Baltimore,Maryland 21218, USA; 4 Materials Science and Engineering, Johns Hopkins University, Baltimore,Maryland 21218, USA

## Abstract

Due to their ability to provide efficient mixing at small scales, confined impinging jet mixers (CIJMs) are employed widely in nanoparticle assembly processes such as flash nanoprecipitation and flash nanocomplexation, which require rapid mixing. In this mixing device, two jets from opposite directions impinge directly on each other forming a thin shear layer that breaks down rapidly into small flow structures. This enables effective mixing of the species transported by each jet by drastically reducing the diffusion distance. In the present study, the mixing performance of a commonly used cylindrical CIJM is examined by direct numerical simulations. Analysis of the simulation results indicates that the interaction of the shear layer with the inner walls of the CIJM is critical in inducing a range of instabilities in the impinging jet flow. By examining flow structures, statistical quantities, and metrics, we have characterized and quantified the mixing quality of a binary mixture in the CIJM. Product uniformity in processes such as precipitation and complexation is expected to depend on the residence time of the constituents, and this quantity is also calculated and compared for the cases with different jet Reynolds numbers. The jet Reynolds numbers of Re = 200, 600, and 1000 are considered, and the simulation results show that the CIJM achieves very good mixing for the Re = 600 and Re = 1000 cases. It is also found that the Re = 600 case performs slightly better than the other cases in terms of uniformity of the residence time. These quantitative analyses offer useful insights into the mechanism of nanoparticle size control and uniformity afforded by the unique flow physics and mixing characteristics in the CIJMs.

## I. INTRODUCTION

In a multi-component reaction or assembly system, effective mixing of different components is critical in controlling the characteristics and uniformity of the manufactured products. Mixing via flow turbulence is highly effective since turbulence rapidly generates flow structures at a much-reduced length scale, where mixing among different components introduced by different flows can occur at a time scale of tens of milliseconds. For chemically reactive systems, a mixing rate that matches or is faster than the reaction rate is important because if the mixing speed is slow, the reaction happens in a temporally and spatially non-uniform manner, resulting in heterogeneous products. In a flash nanoprecipitation (FNP) system, nanoparticles can assemble more uniformly when the average solvent mixing rate is faster than the average phase separation rate of the polymer. Similarly, in a flash nanocomplexation (FNC) system, more uniform nanoparticles can be assembled when the average mixing rate of the polyelectrolytes introduced by the two inlets matches the polyelectrolyte complexation (PEC) rate. Turbulence induced mixing can be achieved by using T connectors,^1^ Tesla mixers, herring-bone mixers,^2^ coaxial jet mixers,^3,4^ confined impinging jet mixers (CIJMs),^5,6^ and multi-inlet vortex mixers (MIVM).^7^

A CIJM consists of two or more impinging jets and a mixing chamber. Liquid chemical solutions are injected as jets into the confined chamber, and the jets impinge inside the chamber. CIJMs are widely used in chemical processes that require fast and thorough mixing and can be used for injecting reactants, such as opposed jet burners^8^ and precipitators.^9^ Rapid breakdown of the shear layer resulting from the impinged jets and transition to turbulence can induce a high degree of mixing of the chemical species even in small-scale mixers, where Reynolds numbers are relatively low [O(1000) or lower].^10^

The study of CIJMs has been driven by extensive industrial need. The initial work of Johnson and Prud’homme^5^ drew significant interest into the mixing and assembly of nanoparticles using CIJMs. Santos *et al.*^11^ introduced small-scale CIJMs into the production of PEC nanoparticles in a continuous and scalable manner. Nikoubashman *et al.*^12^ implemented a CIJM to achieve rapid micro-mixing of polymers with a non-solvent for the directed assembly of soft nanoparticles, demonstrating that this mixing mechanism is highly promising for the mass production of uniformly sized colloidal particles, with a wide variety of polymeric feed materials.

Although CIJMs are being used widely in the industry, understanding of the flow physics and mixing characteristics of the CIJM is still limited. Tucker and Suh^13^ used flow visualization to show that the flow in directly opposed jets transitions at a Reynolds number of ∼140 with the jet velocity as the characteristic velocity scale and the inlet diameter as the characteristic length scale. Wood *et al.*^14^ investigated the flow field in a CIJM experimentally and numerically. They quantified the Strouhal number as a function of Reynolds number and observed a low frequency fluctuation pattern of the flow at a Strouhal number based on the inlet jet diameter and inlet velocity of ∼0.01. Unger and Muzzio^15^ implemented a laser-induced fluorescence (LIF) technique to visualize the concentration field of chemical species injected from one side of a CIJM and offered a technique to better quantify the mixing. Johnson and Wood^10^ quantified the fluctuation frequency of the self-sustained oscillations by using spectral analysis on laser Doppler anemometric measurements. Their results were similar to those of Wood^14^ in that the Strouhal number of the lateral velocity was found to be of the order of 0.01. Santos *et al.*^16^ visualized the flow field experimentally using particle image velocimetry (PIV) and quantified the intensity of turbulence by calculating the root-mean-square (rms) and probability density function (PDF) of the velocity as a function of Reynolds number. Icardi *et al.*,^17^ Santos *et al.*,^18^ and Marchisio^19^ also performed a large-eddy simulation (LES) of the flow in a CIJM.

In the previous studies, the mixing quality in the CIJM was characterized by the intensity of segregation (IOS) and the mixing length scale. The intensity of segregation (IOS), which is defined as the spatial rms of the concentration of chemical species, is often used to quantify the uniformity of the distribution of the chemical species. Wood *et al.*^14^ and Fonte *et al.*^20^ demonstrated that the CIJM has a very good mixing quality in terms of IOS. The IOS of the CIJM was less than 0.1, indicating that the chemical species was distributed almost uniformly. The mixing length scale is also used to quantify the mixing quality, and Lee *et al.*,^21^ Baldyga and Bourne,^22^ and Tucker and Suh^13^ separately made predictions of the mixing length as a function of Reynolds number. However, there is no common method to measure the mixing length scale other than capturing the peak wavelength of the spatial velocity spectrum.^23^

While IOS has been widely used for the quantification of mixing uniformity, this metric is not sufficient to quantify the mixing of multiple chemical species. This is because the uniformity of distribution of one chemical species does not necessarily imply that it is well mixed with the other chemical species. Only when the concentration of each chemical species is similar at each location can it be claimed that good mixing between species has been achieved. In addition, mixing quality is not the only concern for CIJMs when it is used for certain chemical reactions and assembly processes. The uniformity of the product in terms of size and/or composition is of great importance for processes such as drug production, since non-uniform product size or composition can result in variation in drug efficacy.^11^ Non-uniform products may often result from a large variability in the residence time of the constituents.

In the present study, therefore, we have performed the direct numerical simulations for the flow and mixing in the CIJM, and the mixing quality is investigated by quantifying the cross correlation of chemical species within the mixer. In addition, we also quantify the residence time of the species in the device as a surrogate for the uniformity of the product size and composition. The jet Reynolds number is a key variable in the current analysis of the CIJM since it represents the effects of the scale on the performance of these mixers. Scale effects are important since the size of the device and/or the jet velocity are key factors that determine the throughput of the system and, consequently, the rate of generation of products from these mixers.

## II. MODEL CONFIGURATION

### A. Geometry

**FIG. 1.**
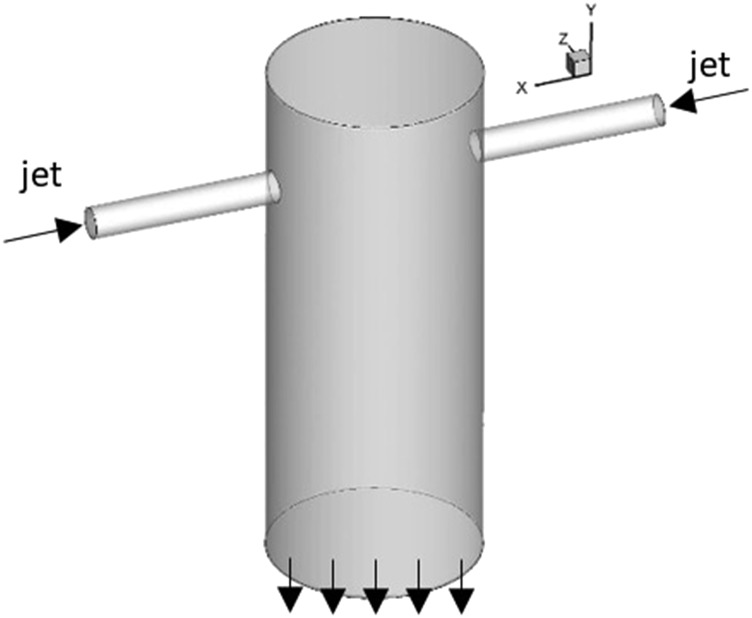
3D view of the CIJM device model used in the current study.

**FIG. 2.**
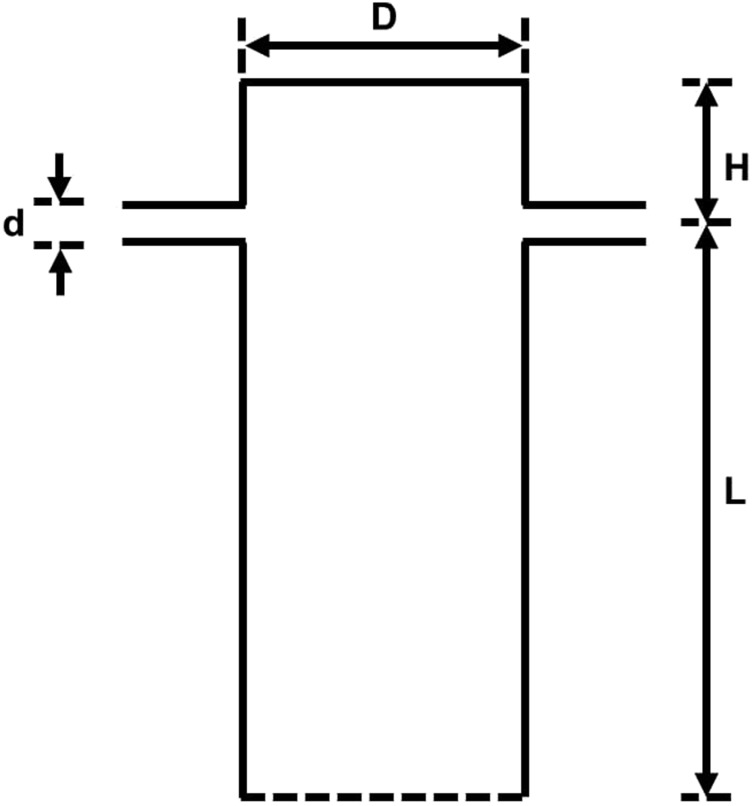
Cross sectional view of the mixer and key dimensions of the mixer geometry.

As shown in Figs. 1 and 2, the confined impinging jet mixer consists of two opposed cylindrical injector tubes (with diameter *d* and length 5*d*) and a cylindrical mixing chamber (with diameter *D*). The injectors are placed closer to the top wall of the mixing chamber with the distance to the top wall designated as *H*. The specific dimension of the geometry used in the present study is extracted from the experimental literature.^20^ Figure 1 shows the three-dimensional (3D) view of the geometry, and Fig. 2 shows the frontal view and dimension of the geometry. The characteristic length scale is chosen to be the diameter of the injector *d*, and the characteristic velocity scale is the average injector (jet) velocity *V*_*in*_. The characteristic time scale is, therefore, defined as t^*^ = *d*/*V*_*in*_. The coordinate system employed in our device is also shown in Fig. 1, and we note that the jets are directed along the *x*-direction, and the axis of the mixing chamber and the outlet is aligned along the *y*-direction. For the present CIJM, *D* = 6.6*d*, the distance between the jet exit and the upper wall (*H*) is equal to 3*d*, and the distance between the jets and the exit (designated as *L*) is 9*d*.

### B. Numerical methods

Flow simulations are performed by solving the incompressible Navier–Stokes equations,

$$\vec{▽}\cdot \vec{u},$$ (1)

$$\frac{\partial \vec{u}}{\partial t}+\vec{▽}\cdot \left(\vec{u}⊗\vec{u}\right)=-\frac{\vec{▽}p}{\rho }+{\nu ▽}^{2}\vec{u},$$ (2)

where $\vec{u}=\left(u,v,w\right)$ is the fluid velocity vector, *p* is the pressure, and *ρ* and *ν* are the density and kinematic viscosity of water. The equation is solved by the projection method^24^ on a Cartesian grid, and a sharp interface immersed boundary method^25,26,27^ is employed to represent the effect of the boundaries. The number of grid points in the x-, y-, and z-directions are 384, 292, and 164, respectively (number of grid points is 18,197,440). The minimum grid spacing is 0.044 *d*. The resolution is chosen based on a grid convergence study (see the Appendix). A fixed time step size of Δ*t* = 0.01*d*/*V*_*in*_ is employed and the maximum *CFL* number (*u*Δ*t*/Δ*x*) is maintained around 0.31 to ensure the stability of the simulation. The uniform, plug flow jet profile is applied at the inlet of the jet tube by the Dirichlet boundary condition (*u* = *V*_*in*_). At the outlet, a zero velocity gradient velocity condition is employed, and the no-slip condition is imposed at the inner wall of the device. The flow simulation is performed on the MARCC (Maryland Advanced Research Computing Center) cluster using 256 CPU cores, and ∼70 h wall time is required for each case to integrate over 400 non-dimensional time (*t*) units. The jet Reynolds number *Re* = *V*_*in*_*d*/*ν* is a key parameter in the analysis of the CIJM and is varied over the range from 200 to 1000 to investigate scale effects in these mixers.

To evaluate the mixing quality of the CIJM, two distinct passively transported scalars are released from the two jets, and the mixing quality of the two scalars is studied. The concentration fields of these scalars are obtained by solving the following convection–diffusion equation:

$$\frac{\partial C_{i}}{\partial t}+\vec{u}\cdot \vec{▽}C_{i}={\frac{1}{Re\cdot Sc}▽}^{2}C_{i};i=1,2,$$ (3)

where *C*_*i*_ is the concentration of *i*th scalar, $\vec{u}$ is the velocity of the fluid, and *Sc* is the Schmidt number of the scalar. The boundary conditions for the scalar are *C*_1_ = 1, *C*_2_ = 0 at the left inlet, *C*_1_ = 0, *C*_2_ = 1 at the right inlet, and *∂C*_*i*_/*∂y* = 0 at the outlet. To investigate the influence of scalar diffusion on mixing, high (*Sc* = 100) and low (*Sc* = 1) Schmidt numbers are considered in this study.

## III. FLOW PHYSICS OF THE CONFINED IMPINGING JET MIXER

The simulations are performed with jet Reynolds numbers of 200, 600, and 1000, and the simulation results are analyzed to gain insight into the flow dynamics and mixing performance.

### A. Vortex dynamics

**FIG. 3.**
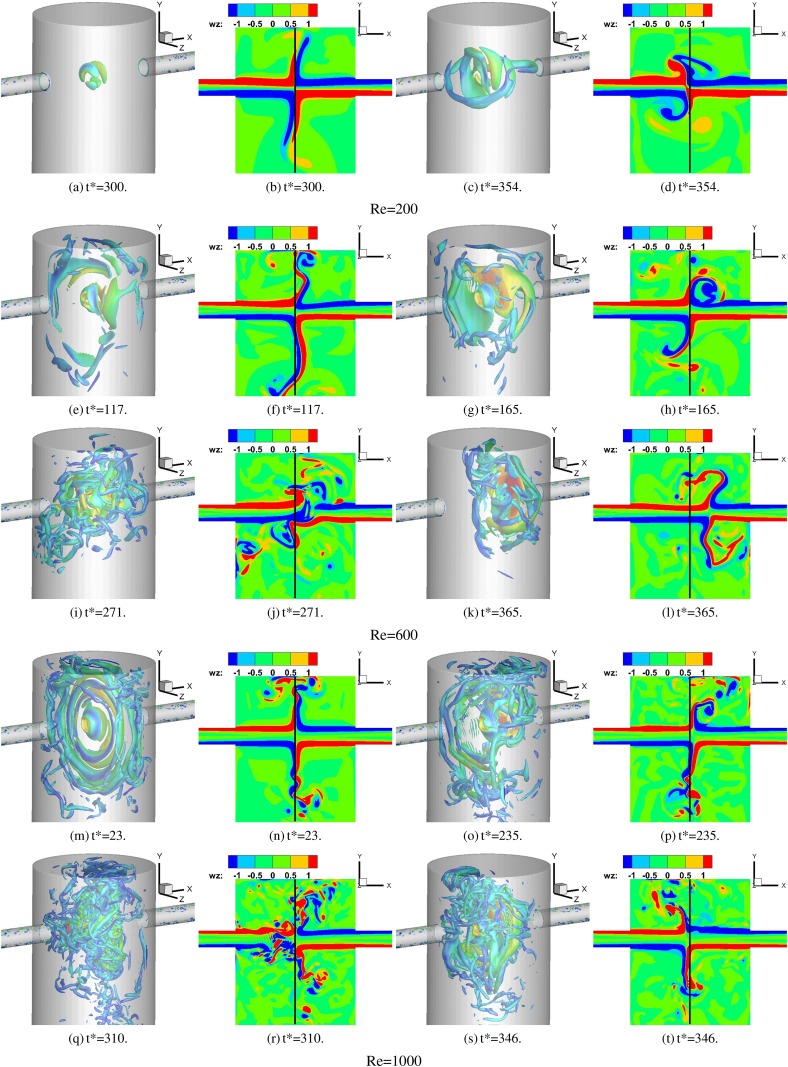
Vortical structures for all three cases at various time instances in the development of the flow. 3D view: Iso-surface of the second invariant of velocity gradient (Q2) colored by velocity magnitude. Cross-sectional view: Z-component vorticity contours on the z=0 plane.

Figure 3 shows the development of a second invariant of the velocity gradient (*Q*) in a 3D view and z-direction vorticity in the side-view slice (the location of the slice is shown in Fig. 2 of the confined impinging jet mixer) for Re = 200, Re = 600, and Re = 1000 cases.

The overall flow patterns in the CIJM with different Re values have some similar features. The two jets directly impinge on each other, and a vortex core and a thin “disk” like shear layer are formed at the impinging center. The vorticity stripes shown in Figs. 3(b), 3(f), and 3(n) signify the shear layer in that slice. Shortly after, the shear layer starts flapping, twisting, and deforming, and small-scale vortex structures are released from the impinging center toward the radial direction of the impinging plane. The experimental result of Fonte *et al.*^20^ also shows an impinging jet induced shear layer, the oscillation of the shear layer, and the vortex shedding induced by the shear layer oscillation, which are similar to our simulation results.

The instability of the flow in the CIJM has several potential mechanisms. First, the shear layer of the impinging jet has intrinsic instabilities, especially at high Reynolds numbers, and this leads to a vortex roll up in the downstream radial direction of the impinging plane. Second, the interaction of the shear layer and the inner walls of the device can also induce the formation of vortex structures. Third, vortex structures generated by the interaction of the jet shear layer and the wall, especially the top wall, can feed perturbations back into the shear layer and even the main jet, inducing a larger amplitude of the fluctuation. The two physical processes, interaction with the inner walls and the feedback to the shear layer of the jet, are coupled with each other, making the flow more and more unstable.

Another interesting phenomenon is that the impingement point, which is defined by the point that has the lowest velocity magnitude $\left(\left|V\right|\right)$ along the *x*-direction centerline of the device, also exhibits large scale stochastic movements. The first source of this movement is that, although the device is symmetrical in the x-direction, the transport of vortex perturbations generates small differences on the left and right sides of the mixer, and this could initiate the movement of the impingement point. The movement of the impingement point away from the center leads to a stronger asymmetry of the vortex distribution and can further drive the movement of the impingement point. The second source is the interaction between the vortex structures and the main jets. This interaction is quite strong and it can induce a sudden shift in the impinging point, as shown in Figs. 3(d) and 3(h). Comparing the flow patterns in the different Reynolds number cases, the most obvious difference is that the vortex structures have a very broad range of scales and the flow is more unstable with increasing Reynolds number.

### B. Flow statistics

**FIG. 4.**
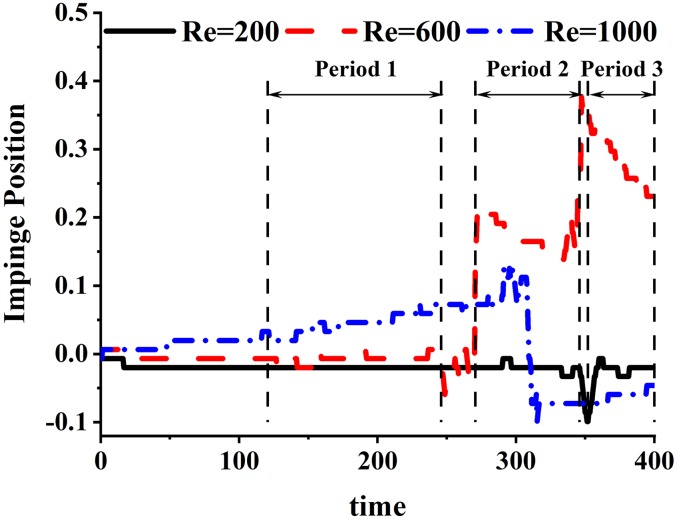
Movement of the impingement point over time for the three cases.

To better evaluate the overall behavior of the flow field, the statistics of the flow field, such as average and fluctuations of the velocity, are examined. In order to average, we need to account for the large changes in the flow associated with the movement of the impingement point. Figure 4 shows a plot of the location of the impingement point for the various cases, and it can be seen that the excursions in the impingement point are the highest for the intermediate Reynolds number of 600, where the impingement point departs significantly from the center of the cylinder, while it is fairly small for Re = 200. A possible reason for this non-monotonic dependence on the Reynolds number is that the interaction between the vortex structures and the main jet for the Re = 600 case is driven by a large scale flow circulation. For the Re = 1000 case, however, this large scale circulation breaks down into smaller scales before it interacts with the shear layer and the main jet. Consequently, the intensity of interaction of the Re = 600 case is stronger, and this results in the larger amplitude of the movement of the impingement point.

**FIG. 5.**
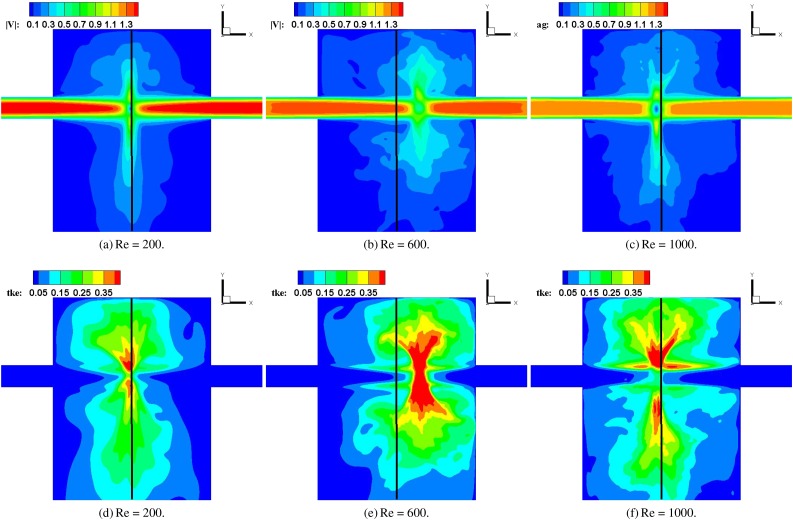
Mean velocity magnitude (top row) and turbulent kinetic energy (bottom row) for the flow along the z = 0 slice for the three cases. The solid lines correspond to the central axis of the mixer.

Given this behavior, the time periods for the accumulation of statistics are divided into several segments, as shown for the Re = 600 case in Fig. 4 and detailed for all the cases in Table I. We obtain time averages over each of these periods and compute the fluctuations about these averages. Figure 5 shows the time-averaged turbulent kinetic energy $\left[\text{defined as TKE}=\frac{1}{2}\left(\bar{{\left(u^{\prime }\right)}^{2}}+\bar{{\left(v^{\prime }\right)}^{2}}+\bar{{\left(w^{\prime }\right)}^{2}}\right)\right]$ in the CIJM for different Reynolds numbers on the side-view plane during the last flow period shown in Fig. 4.

**TABLE I.** Temporal and spatial variation of the jet impingement point. The location of the impingement point is normalized by *D*/2.

Re	Period	Impingement Point	Time Start	Time End
200	Period 0	0.000	0.00	280.67
	Period 1	−0.066	280.67	400.00
600	Period 0	0.000	0.00	120.67
	Period 1	0.022	120.67	246.67
	Period 2	0.590	270.67	346.67
	Period 3	0.947	352.00	400.00
1000	Period 0	0.000	0.00	153.33
	Period 1	0.211	153.33	306.67
	Period 2	−0.222	310.67	400.00

Comparing the flow patterns for the Re = 200, 600, and 1000 cases [Figs. 5(a)–5(c)], we find that the average flow pattern is similar except for the impingement point location. The large scale fluctuations in the flow are driven mainly by the flapping of the impingement disk. The spatial range and amplitude of this flapping can be estimated from the region with high turbulent kinetic energy (TKE), and for all the cases, the flapping region is a fan-shaped region in the two-dimensional (2D) view. Focusing on the region with a high TKE (TKE > 0.2), the spatial range of the flapping for the Re = 200 case is smaller than that for Re = 600 and Re = 1000 cases, while the two latter cases are similar.

**FIG. 6.**
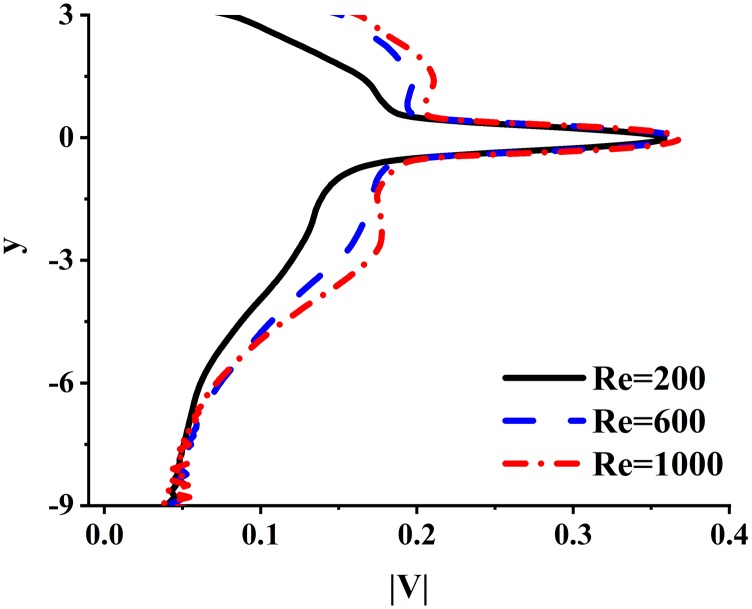
Variation of the temporal and plane-averaged velocity magnitude |*V*| along the vertical direction.

**FIG. 7.**
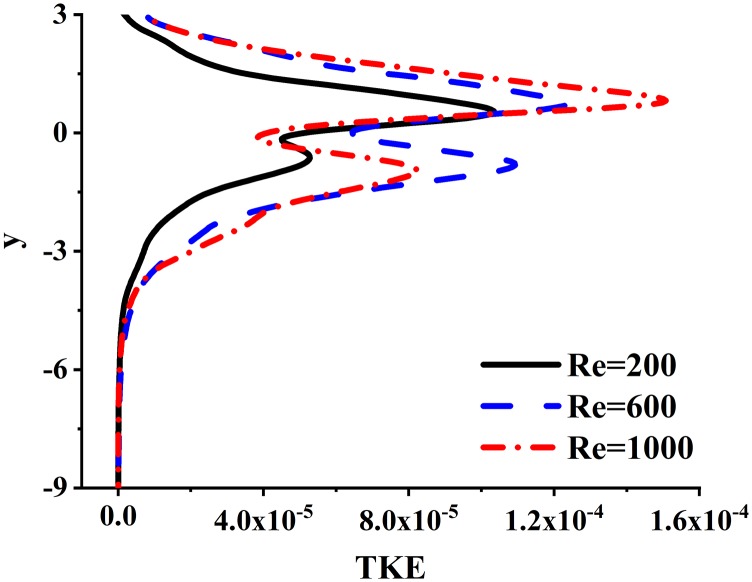
Variation of the temporal and plane-averaged turbulent kinetic energy *TKE* along the vertical direction.

Figures 6 and 7 show the distributions of the spatial and temporal averaged velocity magnitudes, $\bar{\left|V\right|}$, and the turbulent kinetic energy, $\bar{TKE}$, of the CIJM, which are defined as

$$\bar{\left|V\right|}=\frac{\overset{N}{\underset{n=1}{\sum }}\left(\iint_{S}|V{|}_{n}\;dS\right)\;T_{n}}{S\;\overset{N}{\underset{n=1}{\sum }}T_{n}}$$ (4)

and

$$\bar{TKE}=\frac{\overset{N}{\underset{n=1}{\sum }}\left(\iint_{S}TKE_{n}\;dS\right)\;T_{n}}{S\;\overset{N}{\underset{n=1}{\sum }}T_{n}},$$ (5)

respectively, where |*V*|_*n*_ and *TKE*_*n*_ are the velocity magnitude and the turbulent kinetic energy evaluated for each individual flow period, *N* is the number of the flow period, and *T*_*n*_ is the time span of this flow period. |*V*|_*n*_ and *TKE*_*n*_ are integrated for each y-plane of the CIJM.

For each Reynolds number, the distributions of the velocity magnitude are similar; the velocity magnitude at the impingement point (y = 0) is high because of the existence of inlet jets; it drops rapidly away from the impingement point and exhibits a plateau in the region of the fluctuating shear layer. Beyond that, it decreases and reaches a minimum at the outlet of the CIJM.

The distribution of turbulent kinetic energy is quite different from that of the velocity magnitude. The TKE exhibits a minimum at the location of the impingement point and shows two peaks on either side of this minimum. These maxima are associated with the flapping and breakdown of the disk-shaped shear layer, and the peak above the impingement point is higher than the peak of the TKE below. This clearly indicates that the confinement due to the top wall enhances the instability in the shear flow emanating from the jet impingement point. Comparing the TKE distributions for the various Reynolds number cases, the amplitudes of TKE for Re = 600 and Re = 1000 cases are similar, and they are higher than those for the Re = 200 case, which is consistent with the result shown in sub Figs. 5(d)–5(f).

## IV. MIXING QUALITY

### A. Distribution of chemical species

**FIG. 8.**
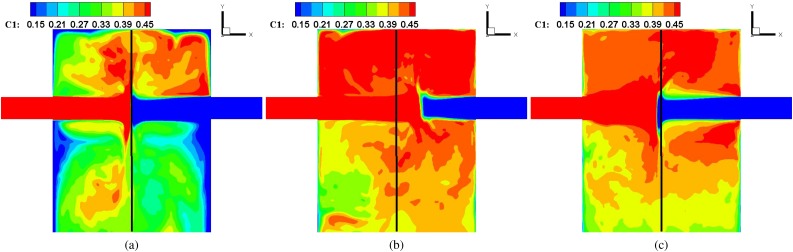
Time-averaged concentration of species 1, *C*_1_ on the z = 0 plane at Sc = 100 for (a) Re = 200, (b) Re = 600, and (c) Re = 1000. The solid lines correspond to the central axis of the mixer.

The mixing process of the CIJM is simulated by continuously releasing a passive scalar at each inlet. Figure 8 shows the time-averaged concentration field of the scalar injected from the left jet (*C*_1_) on the z = 0 plane at Sc = 100 for the Re = 200, 600, and 1000 cases. The scalar is concentrated at the jet and the impingement shear layer, and when the shear layer breaks down into small-scale vortices, the concentration of the scalar rapidly diffused. For all cases, the scalar concentration is higher (close to 0.5) in the region between the inlet jet and the top wall of the mixing chamber. The reason for this is that the recirculating flow between the inlet jet and the upper wall traps the species and makes it more concentrated in this region. Below the jets, the chemical species are transported down and diffused, resulting in a lower concentration of about 0.3. At Sc = 100, one can clearly see that the concentration distribution near the exit is more uniform for the higher Re case by the mixing driven by the small-scale vortices.

### B. Quantitative analysis of the mixing quality

In the CIJM, the species injected into the mixing chamber are diluted and diffused, and their concentrations are decreased. In the previous studies, the spatial uniformity of the concentration has often been employed to quantify the mixing quality. For a binary mixture in the chamber, however, a good quality of mixing and reaction between the species can be achieved if the spatial distributions of the two species are highly correlated. In this study, therefore, the mixing quality in the CIJM is evaluated by the spatial, cross correlation between the two scalar concentration distributions. The mixing index based on the cross correlation, *MI*, is defined as follows:

$$MI\left(y\right)=\frac{\bar{C_{1}^{\prime }\left(x,y,z\right)\cdot C_{2}^{\prime }\left(x,y,z\right)}}{\sqrt{\bar{C_{1}^{\prime 2}}\cdot \bar{C_{2}^{\prime 2}}}},$$ (6)

where $C_{1}^{\prime }$(*y*) and $C_{2}^{\prime }$(*y*) are the variations from the plane-average value of scalar concentration along the length of the mixer, defined as $C_{1}^{\prime }\left(x,y,z\right)=C_{i}\left(x,y,z\right)-\bar{C_{i}}\left(y\right)$, where the bar denotes the spatial average over each y-plane, and *MI* is scaled by the product of the spatial root-mean-square of the scalar concentrations of the two scalars. This metric quantifies how similar the variations in the concentrations of the two scalars are on every vertical plane in the mixer. The more similar the variation of these concentrations, the better the mixing and closer to unity *MI* would be. This metric is, thus, well suited to quantify the mixing in a dilute, binary mixture.

**FIG. 9.**
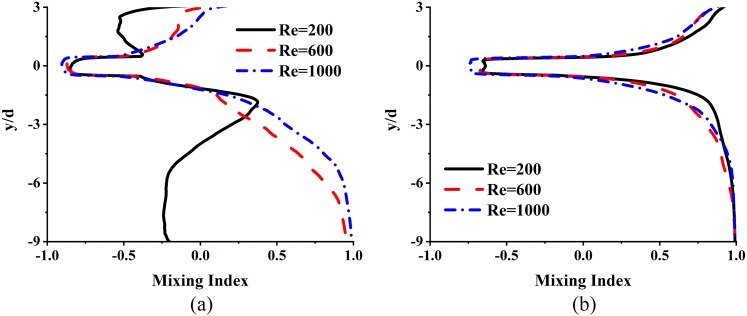
Variation of mixing index based on the cross correlation, *MI* for (a) Sc = 100 and (b) Sc = 1.

The cross correlation metric, *MI*, is evaluated on each y-direction slice of the device and shown in Fig. 9 for Schmidt numbers of 100 and 1. It is observed that for *Sc* = 100, the *MI* in the region above the jets is negative in all cases, mainly because the concentrations there are close to 0.5. Below the jets, the correlation for the Re = 200 case is quite different from that for Re = 600 and 1000 cases. For these latter cases, the *MI* rapidly increases to a value of nearly 1 near the exit, indicating that the spatial distributions of two scalars are almost identical, and thus, they would be well mixed. For the low Schmidt number case (Sc = 1), however, all cases show good mixing both above the jet and near the exit. This is clearly due to the dominant role of molecular diffusion compared to the Sc = 100 case.

## V. RESIDENCE TIME

CIJMs are not only used for mixing but also for the chemical reaction of solutions and complexation of nanoparticles. For instance, Mao and co-workers^11,28^ employed a CIJM for the complexation of plasmid DNA (pDNA) and linear polyethyleneimine (lPEI) and achieved continuous production of lPEI/pDNA nanoparticles. The mixing quality of the CIJM is important for such processes because better mixing can create more uniform local conditions for the reaction, and it is easier to control the size and composition of the product. In addition to local mixing conditions, the uniformity of the product (i.e., the nanoparticle) in terms of size and composition is also important for this kind of continuously occurring chemical reaction. This size and composition depend not only on the local mixing quality but also the overall residence time of the constituents in the mixing chamber. A longer residence time can lead to continuous complexation and growth in particle size, and vice versa. Thus, to achieve a uniform particle size and composition, it is important that the residence time of the constituents in the mixer be as uniform as possible.

Since in the current study we do not model the complexation process, we use the Eulerian flow residence time at the outlet plane of the device as a surrogate metric for product uniformity. The Eulerian flow residence time is the measure of how long the fluid particle resides in the control volume and is computed by the following transport equation:

$$\frac{\partial \tau }{\partial t}+\vec{u}\cdot \nabla \tau =H\left(\vec{x}\right),$$ (7)

where *τ* is the residence time, $\vec{u}$ is the local velocity of the fluid, and $H\left(\vec{x}\right)$ determines the condition of residence inside the chamber, i.e., $H\left(\vec{x}\right)=1$ for locations inside the chamber and $H\left(\vec{x}\right)=0$ for locations outside the chamber. This metric is independent of Schmidt number, since it is for the fluid particle. The residence time is non-dimensionalized by $L/{\bar{V}}_{outlet}$, where *L* is the vertical distance between the inlet jet and the outlet plane, ${\bar{V}}_{outlet}$ is the averaged velocity at the outlet plane $\tau^{*}=\tau {\bar{V}}_{outlet}/L$, and, thus, τ^*^ ≈ 1 corresponds to a straight fluid particle path from the inlets to the exit.

A higher Eulerian residence time indicates that the chemical species or the particles transported by the fluid reside for a longer time inside the chamber before they exit. The underlying hypothesis here is that a fluid particle that resides longer in the chamber will continue to undergo the reaction or complexation and grow in size. Thus, the size of the product and the composition of the nanoparticles resulting from the reaction or complexation process depend directly on the residence time, and therefore, the uniformity of the residence time is related to the uniformity of the product size and composition.

**FIG. 10.**
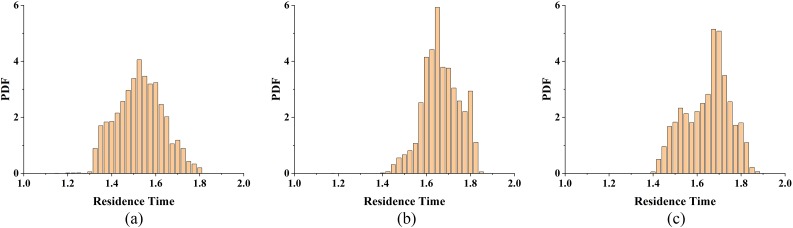
Probability density function (PDF) of the residence time for (a) Re = 200, (b) Re = 600, and (c) Re = 1000. The integral of the PDF has been normalized to unity.

Figure 10 shows the PDF of the residence time at Sc = 100 at the outlet plane of the CIJM, and the mean values and standard deviations for the residence time are shown in Table II. A residence time PDF with a single peak and zero standard deviation would be ideal for generating a uniform size and/or composition of the product for a continuously occurring process such as complexation. It can be noted from Table II that the average residence time for the three cases is about 1.62, indicating that the average distance traveled by fluid particles is ∼1.62 *L*, which is slightly longer than the total length of the mixer. The mean value of the residence time does, however, increase slightly with the Reynolds number. This is because the higher Reynolds number cases have more chaotic flow motions, and fluid particle tracks are more complex and circuitous paths resulting in longer residence times. In terms of the narrowness of the PDF, the Re = 600 case is better than the Re = 1000 and Re = 200 cases since it has a smaller standard deviation. While it is unclear as to what feature of the flow is responsible for this enhanced performance, it could be connected to the intense flapping of the disk-like shear layer and/or the movement of the impingement point. A key takeaway from these results is that the performance of the CIJM is driven by multiple mechanisms, and this can generate a non-monotonic performance with the Reynolds number.

**TABLE II.** Statistics of the residence time.

Re	200	600	1000
Mean value	1.540	1.661	1.680
STD	0.106	0.084	0.101

## VI. CONCLUSIONS

In this study, the flow pattern, turbulence characteristics, mixing quality, and residence time of a confined impinging jet mixer (CIJM) have been investigated via direct numerical simulations. To study the effects of the scale, three Reynolds numbers of 200, 600, and 1000 are examined. Passive scalars are released from the inlets of the mixer, and the mixing quality of the CIJM is characterized. The fluid dynamics in the CIJM is characterized by a number of identifiable features, the first of which is a “disk” like shear layer that is formed by the impinging jets. Vortex shedding is triggered by the intrinsic instability of the impinging jet, and the interaction of vortex structures and the inner wall of the CIJM can enhance the instability of the flow. Large scale circulatory flows can feed disturbances back to the impinging disk as well as the main jet, leading to large, rapid, and highly stochastic movements in the jet impingement point. These movements in the impingement point may induce additional mixing, and the results show that the CIJM achieves very good mixing for the Re = 600 and Re = 1000 cases. The uniformity of the reaction product in these CIJM reactors may depend on the uniformity of the residence time at the exit of the mixer, and the simulations show that the Re = 600 case performs best in terms of this metric. The current study suggests a variety of flow mechanisms that could be exploited to improve the performance of these mixers.

## Figures and Tables

**FIG. 11.**
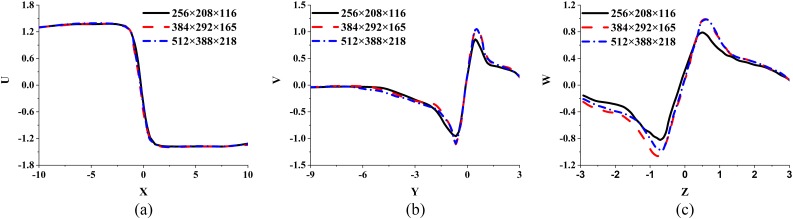
Mean flow velocity for the Re = 1000 case. (a) $\bar{U}$ along the x-centerline. (b) $\bar{V}$ along the x-centerline. (c) $\bar{W}$ along the x-centerline.

## APPENDIX: GRID CONVERGENCE STUDY

A grid convergence test was conducted as a precursor to selecting the grid resolution for the study. The baseline grid was (380 × 292 × 165), and a coarser (256 × 208 × 116) and a finer (512 × 388 × 218) grid were employed for the Re = 1000 case to examine grid sensitivity. The time averaged x-, y-, and z-direction velocity components are shown in Fig. 11. Results with a three grid resolution of the u-velocity along the x-centerline, the v-velocity along the y-centerline, and the w-velocity in along the z-centerline are compared for the three grids, and reasonable agreement (less than 5% difference) in the results is achieved. Given this, we employ the baseline grid for all other simulations.
